# A Restructured Hospital Into a One-Building Organization for COVID-19 Patients: A Resilient and Effective Response to the Pandemic

**DOI:** 10.3389/fpubh.2022.709848

**Published:** 2022-05-24

**Authors:** Simon Bessis, Aurélien Dinh, Sylvain Gautier, Benjamin Davido, Jonathan Levy, Christine Lawrence, Anne-Sophie Lot, Djamel Bensmail, Célia Rech, Muriel Farcy-Afif, Frédérique Bouchand, Pierre de Truchis, Jean-Louis Herrmann, Frédéric Barbot, David Orlikowski, Pierre Moine, Christian Perronne, Loïc Josseran, Hélène Prigent, Djillali Annane

**Affiliations:** ^1^Department of Infectious Diseases, Raymond-Poincaré Hospital, Assistance Publique Hôpitaux de Paris, Paris, France; ^2^UFR Simone Veil, Paris-Saclay University, Montigny-le-Bretonneux, France; ^3^Department of Public Health, Raymond-Poincaré Hospital, Assistance Publique Hôpitaux de Paris, Paris, France; ^4^Department of Physical and Rehabilitation Medicine, Raymond-Poincaré Hospital, Assistance Publique Hôpitaux de Paris, Paris, France; ^5^Microbiology Laboratory and Hygiene, Raymond-Poincaré Hospital, Assistance Publique Hôpitaux de Paris, Paris, France; ^6^Department of Medical Informatics, Raymond-Poincaré Hospital, Assistance Publique Hôpitaux de Paris, Paris, France; ^7^Department of Pharmacy, Raymond-Poincaré Hospital, Assistance Publique Hôpitaux de Paris, Paris, France; ^8^INSERM CIC1429, Clinical Investigation Center, Raymond-Poincaré Hospital, Assistance Publique Hôpitaux de Paris, Paris, France; ^9^Intensive Care Unit, Raymond-Poincaré Hospital, Assistance Publique Hôpitaux de Paris, Paris, France; ^10^Department of Physiology, Raymond-Poincaré Hospital, Assistance Publique Hôpitaux de Paris, Paris, France

**Keywords:** COVID-19 outbreak, preparedness, infection control, emerging infection, resilience

## Abstract

The COVID-19 pandemic is a unique crisis challenging healthcare institutions as it rapidly overwhelmed hospitals due to a large influx of patients. This major event forced all the components of the healthcare systems to adapt and invent new workflows. Thus, our tertiary care hospital was reorganized entirely. During the cruising phase, additional staff was allocated to a one-building organization comprising an intensive care unit (ICU), an acute care unit, a physical medicine and rehabilitation unit, and a COVID-19 screening area. The transfer of patients from a ward to another was more efficient due to these organizations and pavilion structure. The observed mortality was low in the acute care ward, except in the palliative unit. No nosocomial infection with SARS-CoV-2 was reported in any other building of the hospital since this organization was set up. This type of one-building organization, integrating all the components for comprehensive patient care, seems to be the most appropriate response to pandemics.

## Introduction

Official statements by the Chinese government to the World Health Organization reported the first confirmed case of an emerging disease presenting like interstitial pneumoniae in Wuhan, Hubei province, on the 8th of December 2019. The causative agent, secondarily identified as SARS-CoV-2, is responsible for the coronavirus disease 2019 (COVID-19) (1). The virus spread incredibly rapidly all over the world and, as of the 14th of March 2022, has caused 456,908,767 confirmed cases of COVID-19, including 6,041,077 deaths [European Control Disease Center (ECDC) survey (2)]. In France, up to the 14th of March, 23,565,274 confirmed cases, including 141,054 deaths were reported (3).

The COVID-19 pandemic is a unique crisis in modern history per its scale and fast dissemination, challenging numerous institutions: the healthcare, but also economic and political (4, 5) systems. A large influx of patients quickly overwhelmed the organizations in both community and hospital settings. This major event forced all the components of the healthcare systems to adapt and invent new workflows in order to manage an unforeseen number of contagious patients needing isolation. In this context, our tertiary care hospital was at risk of overloading and had to promptly increase its bed capacity to hospitalize more patients.

The Raymond Poincaré hospital is a 386-bed university hospital in the Paris area, belonging to the Greater Paris University Hospital [Assistance Publique Hôpitaux de Paris (APHP)], with a pavilion-like design. It was designated by the French Ministry of Health since the H1N1 crisis in 2009 as a second line center in epidemic risk management during pandemics. Thereafter we were activated for the management of patients suspected of being infected with Ebola virus (2014) and later with MERS-Cov (2015). Our structure was audited and validated by a European commission (EURO NHID) on epidemic risk management in 2014 (6–8). Thus, it became the referral center in the Hauts-de-Seine County (176 km^2^ and 1.6 million inhabitants) close to Paris, for the management of COVID-19 patients as decreed by the Ministry of Health.

On the 24th of February of 2020, the first patient with suspected COVID-19 was hospitalized in a specific ward of the infectious disease department of the hospital, which was separated from the rest of the department by a two double-door entrance. This COVID-19 ward was dedicated to admit all suspected or confirmed COVID-19 patients. In the event of case of ruled-out COVID-19 diagnosis (negative SARS-CoV2 PCR assay), the patients would be relocated to the usual wards. Overall, 27 patients with confirmed COVID-19 were hospitalized in this ward until the 18th of March 2020, and on this date we dedicated the entire building to the management of confirmed COVID-19 patients.

We aim to describe the reorganization of structural, functional and management setups that occurred in our tertiary care hospital during the COVID-19 pandemic first wave.

## Organization of COVID-19 Pavilion

### Usual Organization

**Figure 1:**
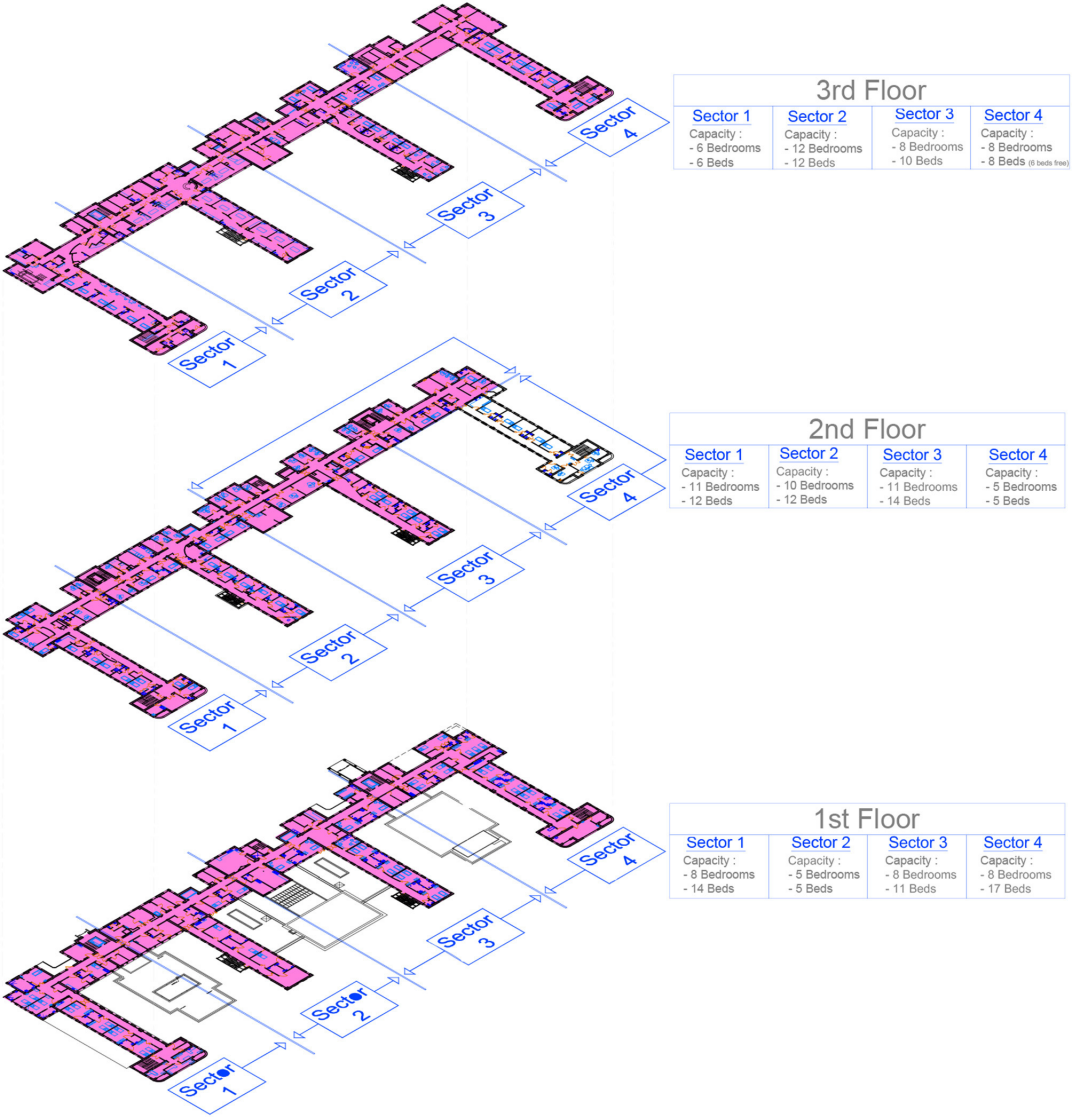
Detailed plan of the COVID building units (in pink color, units dedicated for COVID).

The pavilion is a four-floor structure which ordinarily includes the following departments: an outpatient consultation area, a gymnasium for rehabilitation and the pharmacy department on the ground floor; a 42-bed physical medicine and rehabilitation (PMR) department on the first floor; a 26-bed infectious disease department on the second floor; and a 15-bed intensive care unit (ICU) on the third floor (Figure 1).

### COVID-19 Pavilion Organization

Several steps were taken to reorganize the entire pavilion.

Access to the COVID-19 pavilion was provided through a dedicated access door and lift, accessible only to health professionals working in the pavilion, and the ambulances of the emergency medical assistance service (SAMU and SMUR). This lift connected all departments, including the ICU. The visitors gained access through another dedicated entrance.

At ground floor, the ambulatory screening of community-based suspected COVID-19 patients was carried out by respiratory swab, in a consultation area with a dedicated entry access. Screening was carried out from 9:00 a.m. to 1:00 p.m., and tests results were available in the afternoon. Follow-up visits were also organized for the patients treated in our center for a medical reassessment after hospital discharge, only in the afternoon to avoid potential contaminations. There were also a gymnasium dedicated to respiratory and physical rehabilitation for COVID patients, the pharmacy department, and a resting area for healthcare workers (HCW) available seven days a week.

On the first floor, four units were composed of respectively 14 beds, 5 beds, 11 beds, and 17 beds (so a total of 47 beds). Units 1 and 2 (19 beds) were dedicated to acute care, unit 3 (11 beds) to post-ICU respiratory rehabilitation, and unit 4 (17 beds) to the rehabilitation of patients. Two beds in unit 2 were dedicated to palliative care. The management of patients was carried out by the rehabilitation physicians under the supervision of infectious diseases specialists (IDS), except for the rehabilitation unit.

On the second floor a unit of 12 beds was opened, bringing the total number of acute care beds to 38. A reserve of five potential supplementary beds was also organized in another unit, with the possibility of cohorting patients in double rooms if the influx of patients was too important, increasing the number of supplementary beds to ten. COVID-19-free patients were transferred to two other buildings.

In the third floor, the ICU was entirely dedicated to the management of severe COVID-19 patients. Its capacity of high dependency beds increased from 15 to 32 beds in 6 days (from the 18th to the 24th of March). A COVID-19 free ICU unit was activated in another building.

### Patient Pathway

With the presence on site of a call and intervention center for the emergency medical assistance service (SAMU and SMUR), a direct telephone line was set up with an integrated delivery system (IDS) for better triage and management of patient transfers to our center.

**Figure 2:**
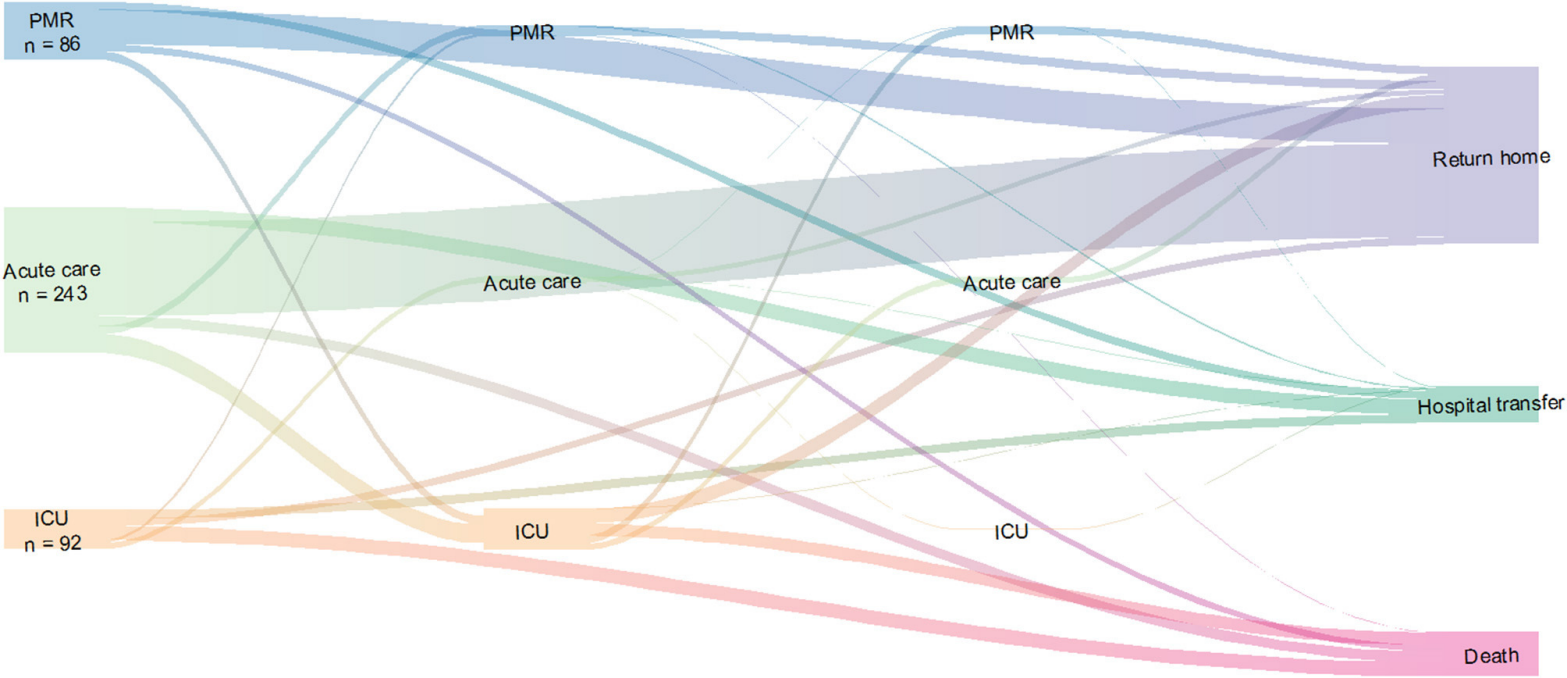
Patients pathway in COVID-building during the pandemic. The thickness of the lines is proportional to the size of the patients' flow. This flow diagram does not take into account the temporality. It describes the trajectory of the patients according to the passages in the different departments. PMR, physical medicine and rehabilitation; ICU, intensive care unit.

At the beginning of the crisis, a reflection was initiated to manage COVID-19 infected patients and optimize patient pathway. Starting at their admission to our hospital, the patients could receive complete care in the same building: acute care, ICU, respiratory weaning unit, rehabilitation or palliative care if needed. We provide a graph of the patient flow between different wards in the same building during this period using the hospital database and data from the Public Health department (Figure 2).

At last, in case of death, a specific unit on the second floor was dedicated to the presentation of the body to relatives and friends following the current regulation during the pandemic: no more than two people at the same time and transmission-based precautions, for no more than one hour. Psychological support was available seven days a week and 24 h a day.

Thus, COVID patients did not need to be outside of the building except for radiological exams.

## Staff Resources

During this pandemic, three phases could be distinguished: A set-up period from the 2nd to the 18th of March 2020, a cruising phase from March 19th to April 12th 2020, and a landing phase from April 13th until 29th of June 2020.

**Figure 3:**
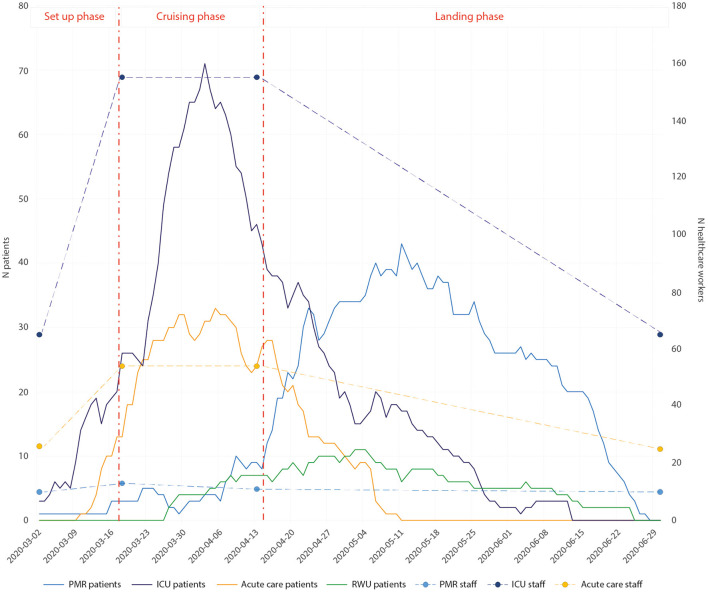
Visualization of patient numbers by date and the three phases of the crisis. The healthcare staff in intensive care and acute care are informed at the start of each phase and a trend in staff numbers is reported for each phase. PMR, physical medicine and rehabilitation; ICU, intensive care unit; RWU, respiratory weaning unit.

During the cruising phase, additional staff was allocated to the pavilion from other in-hospital departments or from other establishments, resulting in a 42% increase in the ICU, a 50% increase in acute care wards, and a 20% increase in the PMR unit. Figure 3 shows the number of patients and staff resources over time in each unit.

Dedicated COVID meetings were set up every morning with the medical doctors, paramedics and rehabilitation therapists of the building in order to take stock of the beds, to organize ethical discussions for the resuscitation of patients, and to decide therapeutics. This allowed the coordination of all the efforts of the different actors involved in the care of COVID-19 patients.

Two senior physicians were dedicated to the consultation area for ambulatory screening in the morning and follow-up visits of patients who recovered from COVID-19 in the afternoon. In acute care wards, the reorganization of human resources allowed the deployment of two residents and a senior physician per unit of 12 to 14 beds. A list of on-call duty was systematically drawn up, composed of a senior physician and a resident during nights and weekends. In the ICU, there was a major staff reinforcement with 7 senior physicians, 8 residents and 35 paramedical staff, with two physicians on duty during nights and weekends.

During the pandemic, HCW were forced to adapt their protection measures on several occasions, in particular to cope with the lack of equipment. In addition to washing their hands with a hydro-alcoholic solution between each treatment, all staff had to wear disposable clothing and change it every day. Surgical masks had to be worn throughout the day (and changed every six hours) within the building. HCW were required to put on an over-blouse, gloves, protective eyewear and a head covering before entering a patient's room, and to remove them when leaving the room.

## Restructuring COVID-19 Departments

To provide optimal care during this epidemic, a coordinated and multidisciplinary multi-professional management is of paramount importance between the ICU, the departments of infectious diseases (ID), of physical medicine and rehabilitation (PMR), of Infection Prevention and Control (IPC), public health specialists, and the hospital direction (9).

### ICU

Early in the process, admission criteria for the ICU were collectively defined and reevaluated over time.

The most severe patients presenting COVID-19 required intensive care for over two or three weeks (10), mainly because they required mechanical ventilation. According to our local guidelines, patients in the ICU on mechanical ventilation were tracheotomized within three days by a mobile Ear Nose Throat team.

Patients in the ICU were not allowed to receive any visitor, thus special cell phones were set up for patients to call relatives if needed. Digital and visual interaction via cell phones and tablets were made possible thanks to the involvement of the staff. Psychological support for patients and relatives were provided by dedicated psychologists and trained staff.

### Respiratory Rehabilitation Unit

Early in the crisis, the respiratory weaning unit (RWU) was set-up ex nihilo in seven days on the first floor (11). Its main goal was to limit bed shortage in the ICU. Patients with a stabilized respiratory and hemodynamic status were transferred to the RWU. Two physiotherapists were fully dedicated to motor and respiratory rehabilitation.

### Medico-Technical Units

#### Medical Imagery

Patients were transferred to radiology via specific access tunnels linking the buildings. Two mobile X-ray machines were dedicated to the pavilion for images at the patient's bed without the need to transport them.

#### Microbiology Laboratory

At the start of the crisis, the COVID-19 RT-PCRs were sent via an approved transporter to the national reference center for a result within 8 h. Our virology laboratory later became a dedicated center for carrying out PCR tests, with a result within 4 h and the capacity of 100 PCRs per day. Indeed, the microbiology laboratory was reorganized to dedicate an upgraded to BSL3 standard technical room, solely to the analysis of respiratory samples in a laminar flow chamber. Individual protective equipment identical to that of the caregivers was provided. This reorganization also allowed all the usual biological analyzes to be carried out while maintaining a high level of security for the staff. From April 15th, a reinforcement of equipment and personnel from the scientific gendarmerie allowed to raise the test rate to 1,000 PCR per day, in order to screen outpatients, hospital staff, and patients admitted to the hospital.

### Logistical Units

All waste was treated as coming from care activities with an infectious risk. They were disposed of four times a day by the internal waste disposal service, and transported to the central storage area of the facility before their disposal by a specialized service provider. A dedicated laundry service was set up in the buildings for the staff in charge of COVID patients, allowing them to change their outfit and gown every day.

### Management of Medicines and Health Products

Pharmacy department was strongly involved in the management of the pandemics. Working days and hours were modified, although no additional or temporary staff could be recruited. This led to tense and overwork situations in this department. An additional storage area of 40 m^2^ was freed up and had to be managed, making logistics more complex. Available drugs and medical devices were significantly increased by pharmacy staff, in a qualitative and quantitative manner, on every floor and especially in the ICU. Between mid-March and the end of April, three drug deliveries per day (with 30 order references per day) were necessary to cover drug requirements, in addition to weekly orders, whereas 3 orders a week were sufficient before the pandemic. In parallel, the pharmacist in charge of supplier relationships had to contact their device suppliers every day to ensure on time deliveries.

In the ICU, all available rooms and storage shelves were used to store supplementary drugs, in order to reduce medication error risks. The important size and volume of some medical devices (filter, ventilator circuit...) constrained potential storage. Automated drug storage cabinets (equipment used in half of the units, including the ICU) were no longer suited to this situation, because of great amounts of necessary medications, frequent replenishments blocking access to the cabinet, urgent needs without possibility to wait turn, and lack of time to create new user profiles and train them. A thorough storage control of drugs and devices was performed every day in the ICU and in the pharmacy department. These drugs had to be stored in a conspicuous location, because they were the most at risk of shortage during the pandemic.

A pharmacy technician was dedicated to two daily storage controls of critical medicines, both in the ICU (all storage locations) and in the pharmacy department. Immediate orders to drug suppliers were then placed with adjusted quantities. Physicians were regularly informed of calculations of drug availability for all ICU patients to adapt medication protocols in case of shortage. New medication protocols were developed with training of physicians and nurses to preserve essential drugs such as curares, parenteral benzodiazepines, and to spare syringes. During all the epidemic period, consumption of 160 medical devices increased significantly, and were multiplied by more than 20 times for some respiratory devices like endotracheal tubes, tracheostomy tubes and closed suction systems. Consumptions of curares and sufentanyl were multiplied by 20 times, those of midazolam, dialysis solutions, albumin, propofol, dexmedetomidine and norepinephrine were multiplied by five to 10 times. There was no break in the patients' treatments thanks to continual checks, and daily adjustment of minimal thresholds. At times, substitute devices (filter, closed suction system, swab) had to be proposed in case of shortages. New recommendations in terms of conditions of use for respiratory equipment (nasal prongs, masks) were issued to confirm the lifespan extension of these devices when shortages were too important.

Overall, a new management policy was implemented, regarding supply difficulties: enlargement of suppliers, use of charitable contributions, controlled delivery with data traceability, reinforced cooperation with the hygiene department and directorate of care.

## Coordination and Crisis Management

During the outbreak, the COVID-19 pavilion benefited from significant autonomy regarding decisions about the implementation of a dedicated and specific pathway for COVID patients and the logistical organization (materials and workforce) of the building. The governance of the pavilion was spontaneously organized by mutual adjustment between the different heads of departments. This type of coordination seems to have been possible due to the physical proximity of the teams and the complementarity of the coordinated activities present in the building.

The questions and collective answers were secondarily exposed to the management of the hospital during crisis meetings which were daily organized for the pandemic. These meetings, which took place outside the COVID-19 pavilion, made it possible to easily share with the entire hospital community the decisions taken by the departments and the issues that remained to be solved. Following a decentralization approach contributes to the decentralization of decision-making power in crisis situations and is likely to promote the resilience of health organizations (12). This kind of empowerment promoting participatory strategies allowed greater flexibility and greater responsiveness to the changes.

## Activity Reporting

From the 2nd of March to the 29th of June, a total of 278 COVID-19 patients were hospitalized in the pavilion (see Figure 3). Overall, 92 were in the ICU, 243 in acute care, 86 in PMR, with some patients having multiple stays in multiple units. Twenty-six patients were transferred from acute care to the ICU and 28 from the ICU to acute care. Also, 15 patients were transferred from acute care to PMR. Finally, three patients were transferred to the ICU from other regions to alleviate other hospitals. In the ventilatory weaning unit, 30 patients were managed. This organization allowed ease of transfer between the different units while minimizing the risk of contamination of patients and HCW. We only reached 100% of our capacity for 24 h on the 3rd of April.

Among the 278 patients, 64.0% were male, with a median age of 59.7 years [range 17–98], 39 of them (14.0%) passed away. In the acute care, 243 patients were managed, 59.3% male, and a median age of 59.7 years, while in the ICU, 68.5% were male, and the median age was 58.6 years. The overall median length of stay for the patients was 10.9 days, 12.9 days in the ICU, 6.6 days in acute care, 15.0 days in PMR, and 13.0 days in the ventilatory weaning unit.

Overall, our global hospital mortality rate was 13.3% (37/278) compared to 18.1% in France. The mortality rate in the ICU was 31/92 (33.6%). The mortality rate in the non-ICU medical units was 6/243 (2.4%). Of the patients who died, 62.2% were male, and the median age was 72.0 years.

Within the same period, we screened 512 members of the nursing staff through our ambulatory screening, 129 (25.2%) had a positive PCR, ten were directly hospitalized. Before the one building organization we identified seven nosocomial transmission cases among patients. All of them occurred in other non-infectious disease pavilions, all before the set-up of this new organization.

## Discussion

As many hospitals in France, our structure and organization had to adapt to handle the mass influx of infectious patients, many of them severe. The implementation of a one-building organization with an ICU, acute care, PMR and screening area enabled us to meet the challenges posed by the COVID-19 pandemic. The transfer of patients from a ward to another was easier and faster due to these organizations and our pavilion structure. Excluding the palliative unit, there were only a few deaths in the acute care ward. At last, no nosocomial infection with SARS-CoV-2 was reported in any other building of the hospital since this organization was set up.

The COVID-19 pandemic has presented an unprecedented array of challenges for health care institutions worldwide, through its massive and rapid spread. It required effective and unconventional responses, and innovative ways of collaboration and communication. Because of its severity and high contagiousness, one of the main challenges was the necessity to restructure the usual care models.

Several descriptions of worldwide healthcare delivery organizations that effectively responded to the pandemic through various actions and redesigns (13) showed that the COVID-19 pandemic could be used as an opportunity to prepare for a possible next pandemic.

Firstly, to counteract the massive patient volume and the extreme need for ICU beds and ventilators, many hospitals had to expand their critical care capacity. For instance, the New York City Health + Hospitals had their number of ICU patients increase by 3-fold (14) during the first wave. Furthermore, to prevent staff shortages within the New York City Health and Hospitals Corporation, staff from their various facilities was redeployed to the emergency departments and ICUs (15). Additional HCW were also recruited from other health care structures (15, 16).

Additionally, COVID task forces were created in several hospitals to help with patient triage and implement protection measures guidelines, such as at the University of California, San Francisco (UCSF) (16).

Another French hospital implemented hospital-wide communication through their intranet, conferences open to HCW, and video interviews with experts from different hospital departments (17).

Some limits were raised at our hospital, such as the low number of elevators which were insufficient to properly separate patient and logistical pathways, and the absence of radiology in the building.

Furthermore, our organization would not be applicable to some medical institutions or in other countries, because of various differences in healthcare systems especially in the pre-COVID-19 pandemic era.

Therefore, we believe that this type of one-building organization, integrating all the components for comprehensive patient care, could be an appropriate response to the COVID-19 pandemic and future pandemics. Moreover, management of infectious risk should be integrated in the architecture plans of future hospitals with the possibility to segregate independent units, following strategies described here, in order to upgrade their capacity in the event of massive influx of patients exposed to highly contagious infectious diseases.

## Data Availability Statement

The original contributions presented in the study are included in the article/supplementary material, further inquiries can be directed to the corresponding author.

## Author Contributions

SB, AD, SG, LJ, FB, and DA wrote the first draft of the manuscript. SG conceived the figures. All authors reviewed the manuscript and approved the final version.

## Conflict of Interest

The authors declare that the research was conducted in the absence of any commercial or financial relationships that could be construed as a potential conflict of interest.

## Publisher's Note

All claims expressed in this article are solely those of the authors and do not necessarily represent those of their affiliated organizations, or those of the publisher, the editors and the reviewers. Any product that may be evaluated in this article, or claim that may be made by its manufacturer, is not guaranteed or endorsed by the publisher.

## Appendix

### The Garches COVID 19 Collaborative Group

Department of Intensive Care: Djillali Annane, MD, PhD (1,2,5) Xavier Ambrosi, MD (4) Suzanne Amthor, MD (1) Rania Bounab, MD (1,2) Ryme Chentouh, MD (1) Bernard Clair, MD (1) Abdallah Fayssoil, MD (1,2,5) Diane Friedman, MD (1) Nicholas Heming, MD, PhD (1,2,5) Virginie Maxime, MD (1) Pierre Moine, MD, PhD (1,2,5) Myriam Niel Duriez, MD (1) David Orlikowski, MD, PhD (1,2,5,8) Francesca Santi, MD (1,2)

Pharmacy: Frédérique Bouchand, PharmD (1) Muriel Farcy-Afif, PharmD (1) Hugues Michelon, PharmD, MSc (1) Maryvonne Villart, PharmD (1)

Research Staff: Isabelle Bossard (8) Tiphaine Barbarin Nicolier (1) Stanislas Grassin Delyle, MCUPH (2,3,5) Elodie Lamy (2,5) Camille Roquencourt, MD (5) Gabriel Saffroy (2) Etienne Thevenot (5)

Department of Intensive Care Residents: Baptiste Abbar (1) Steven Bennington (1) Juliah Dray (1) Pierre Gay (1) Elias Kochbati (1) Majistor Luxman (1) Myriam Moucachen (1) Alice Pascault (1) Juan Tamayo (1) Justine Zini (1)

Department of Anesthesia, Perioperative Care, and Pain: Marie Boutros, MD (1) Anne Lyse Bron, MD (11) Denys Coester, MD (12) Etiennette Defouchecour, MD (11) Brigitte Dosne Blachier, MD (11) Léa Guichard, MD (1) Damien Hamon Pietrin, MD, PhD (1) Hakim Khiter, MD (1) Valéria Martinez, MD, PhD (1,2,6) Simone Meuleye, MD (1) Suzanne Reysz, MD (1) Sebastien Schitter, MD (1) Chawki Trabelsi, MD (1)

Pediatric Critical Care Unit: Helge Amthor, MD, PhD (1,2,7) Jean Bergounioux MD (1,2,5) Maud Guillon, MD (1) Amal Omar, MD (1)

Laboratory of Physiology: Frédéric Lofaso, MD, PhD (1,2,7,10) Helene Prigent, MD, PhD (1,2,7,10)

Department of Rehabilitation and Physical Medicine: Djamel Bensmail, MD, PhD (1,2,7,10) Pierre Denys, MD, PhD (1,2,7,10) Charles Joussain, MD, PhD (1) Lauren Kagane, MD (1) Thibaut Lansaman, MD (1) Hélène Le Liepvre, MD (1) Antoine Leotard, MD, MS (1) Jonathan Levy, MD, MS (1,2,7,10) Claire Malot, MD (1) Julie Paquereau, MD (1) Celia Rech, MD (1)

Department of Rehabilitation Residents: Florence Angioni (1) Elsa Chkron (1) Céline Karabulut (1) Jérôme Lemoine (1) Noémie Trystram (1) Julien Vibert (1)

Department of Infectious Diseases: Simon Bessis, MD (1,2) Pascal Crenn, MD, PhD (1,2,7) Benjamin Davido, MD, MS (1) Aurélien Dinh, MD, MS (1,2) Stéphanie Landowski, MD (1) Hélène Mascitti, MD, MS (1) Morgan Matt, MD (1,2) Christian Perronne, MD, PhD (1,2) Véronique Perronne, MD (1) Soline Simeon, MD, MS (1) Pierre de Truchis, MD, MS (1)

Department of Infectious Diseases Residents: Marc Hobeika (1) Louis Jacob (1) Nicolas Kiavue (1) Aymeric Lanore (1) Aurélie Le Gal (1) Julia Nguyen Van Thang (1)

Department of Microbiology and Innovative Biomarkers Platform: Coralie Favier (1) Jean Louis Gaillard, MD, PhD (1,2,5) Elyanne Gault, MD, PhD (1,2,5) Jean-Louis Herrmann, PharmD, PhD (1,2,5) Christine Lawrence, PharmD (1) Virginie Lebidois, PharmD (1) Latifa Noussair, MD (1) Martin Rottman, MD, PhD (1,2,5) Anne-Laure Roux, PharmD, PhD (1,2,5) Sophie Tocqueville (1) Marie-Anne Welti, MD, PhD (1,2,5) And the nonmedical staff of the Department

Department of Laboratory Medicine and Pharmacology: Jean Claude Alvarez, MD, PhD (1,2,5) Mehdi Djebrani, PharmD (1) Pierre-Alexandre Emmanuelli (1) Firas Jabbour, PharmD (1) Lotfi Lahjomri, MD (1) Mathilde Parent, MD (1) And the nonmedical staff of the Department

Department of Radiology: Amine Ammar, MD (1) Najete Berradja, MD (1) Robert-Yves Carlier, MD, MS (1,2,7,14) Annaelle Chetrit, MD (1,2) Caroline Diffre, MD (1,2) Myriam Edjlali, MD, PhD (1,15) Zaki El Baz, MD (1,14) Adrien Felter, MD (1) Catherine Girardot, MD (1,13) Ahmed Mekki, MD, MS (1,2) Dominique Mompoint, MD (1) Dominique Safa, MD (1) Tristan Thiry, MD (1)

Department of Radiology Residents: Margot Armani (1) Olivier de Barry (1) Antoine Kirchner (1) Jeffery Zhou (1)

Department of Forensic Medicine: Geoffroy Lorin de La Grandmaison MD, PhD (1) Department of Forensic Medicine Resident Kevin Mahe (1)

### Affiliations

^1^ Hôpital Raymond Poincaré, GHU APHP, Université Paris Saclay, Garches, France

^2^ Faculté Simone Veil Santé, Université Versailles Saint Quentin en Yvelines, Université Paris Saclay, Montigny-le-Bretonneux, France

^3^ Hôpital Foch, Suresnes, France

^4^ Centre Hospitalier Universitaire de Nantes, Nantes, France

^5^ Université de Versailles Saint-Quentin-en-Yvelines/INSERM, Laboratory of Infection & Inflammation–U-1173, Montigny-le-Bretonneux, France

^6^ Université de Versailles Saint-Quentin-en-Yvelines/INSERM, Centre d'Evaluation et de Traitement de la Douleur–U-987, Boulogne-Billancourt, France

^7^ Université de Versailles Saint-Quentin-en-Yvelines/INSERM, Handicap Neuromusculaire–U-1179, Montigny-le-Bretonneux, France

^8^ Centre d'Investigation Clinique, Garches, France

^9^ Commissariat à l'Energie Atomique, CEA Paris Saclay, Gif-sur-Yvette, France

^10^ Fondation Garches, Garches, France

^11^ Clinique Jouvenet, Ramsay Santé, Paris, France

^12^ Clinique de la Muette, Ramsay Santé, Paris, France

^13^ Polyclinique Mantaise, Mantes-La-Jolie, France

^14^ Centre Hospitalier Intercommunal Poissy/Saint-Germain, GHT Yvelines Nord, Poissy, France

^15^ IMA-BRAIN/INSERM–UMR-1266, DHU-Neurovasc, Centre Hospitalier Sainte-Anne, Paris, France
